# Distributed data processing for public health surveillance

**DOI:** 10.1186/1471-2458-6-235

**Published:** 2006-09-19

**Authors:** Ross Lazarus, Katherine Yih, Richard Platt

**Affiliations:** 1 Channing Laboratory, Department of Medicine, Brigham and Women's Hospital and Harvard Medical School, Boston, MA, USA; 2 Department of Ambulatory Care and Prevention, Harvard Medical School, Harvard Pilgrim Health Care; Harvard Vanguard Medical Associates, Boston, MA, USA

## Abstract

**Background:**

Many systems for routine public health surveillance rely on centralized collection of potentially identifiable, individual, identifiable personal health information (PHI) records. Although individual, identifiable patient records are essential for conditions for which there is mandated reporting, such as tuberculosis or sexually transmitted diseases, they are not routinely required for effective syndromic surveillance. Public concern about the routine collection of large quantities of PHI to support non-traditional public health functions may make alternative surveillance methods that do not rely on centralized identifiable PHI databases increasingly desirable.

**Methods:**

The National Bioterrorism Syndromic Surveillance Demonstration Program (NDP) is an example of one alternative model. All PHI in this system is initially processed within the secured infrastructure of the health care provider that collects and holds the data, using uniform software distributed and supported by the NDP. Only highly aggregated count data is transferred to the datacenter for statistical processing and display.

**Results:**

Detailed, patient level information is readily available to the health care provider to elucidate signals observed in the aggregated data, or for ad hoc queries. We briefly describe the benefits and disadvantages associated with this distributed processing model for routine automated syndromic surveillance.

**Conclusion:**

For well-defined surveillance requirements, the model can be successfully deployed with very low risk of inadvertent disclosure of PHI – a feature that may make participation in surveillance systems more feasible for organizations and more appealing to the individuals whose PHI they hold. It is possible to design and implement distributed systems to support non-routine public health needs if required.

## Background

Timely identification and subsequent reaction to a public health emergency requires routine collection of appropriate and accurate data about the occurrence and location of cases of illness. There is substantial interest in using routinely collected electronic health records to support both the detection of unusual clusters of public health events and the response to public health threats detected by other means. Such data are also useful to reduce an initial alert level, if it is clear that no unusual illness clusters exist in a community. Ideally, such systems operate automatically and include sensitive and specific statistical surveillance software and alerting systems. These are often referred to as syndromic surveillance systems [1,2], because they typically rely on the non-specific signs and symptoms that may provide the earliest evidence of a serious public health threat, such as anthrax or SARS.

Many syndromic surveillance systems gather potentially identifiable, individual patient-level encounter records. These records are typically collected without name or address, but they do contain enough identifiers to allow re-identification in some circumstances. The potential for re-identification is greatest when records are collected from ambulatory settings or health systems that supply a unique identifier that allows the very useful identification of repeated visits over time. The risk of disclosing sensitive information that can be linked to the individual also increases when the health care facility provides more than occasional care.

In the United States, the Health Insurance Portability and Accountability Act [3] (HIPAA) specifically exempts transfer, use and retention of identifiable electronic personal health information (PHI) to support public health activities. This exemption also applies to syndromic surveillance activities, although HIPAA was developed before large volumes of such data concerning individuals who are not suspected of having a reportable condition were being used for public health purposes in the absence of any known public health emergency. Despite the exemption, data providers may be unwilling to offer identifiable data for surveillance purposes in the face of increasing awareness of the potential costs of inadvertent disclosure or inappropriate use of PHI. Additionally, their patients may object to their providing it. These concerns are common to many developed countries and under these circumstances, designs that minimise the risk of inadvertent disclosure may be needed in order to gain the cooperation of data custodians, for surveillance systems to be feasible. The focus of this paper is on one such design, in which initial data aggregation is performed to decrease the risk of any PHI being inadvertently disclosed, before the aggregate data is centralised for subsequent statistical analysis. Although the system we describe is currently operating in the United States and many of the implementation details are specific to that context, some of the conceptual issues we describe and some of the lessons we have learned may be directly relevant to public health practice in other countries.

While it is possible to centrally collate and process de-identified records, there is a potential problem with statistical inference if multiple records from the same individual are not distinguished. This problem arises because many statistical analysis techniques applicable to surveillance, such as Generalised Linear Mixed Models [4] (GLMM), depend on the assumption that observations are statistically independent. Inference based on this assumption using ambulatory care encounter data will likely be biased if the model cannot distinguish observations from multiple encounters during a single course of illness from a single individual patient. Although the extent of this bias has not been quantified, the problem is clearly illustrated by real data. In more than half of the individuals with multiple lower respiratory syndrome encounters over a four year period from one large ambulatory care practice, a second encounter with the same syndrome was noted less than 21 days after the first encounter [1]. Our approach to this problem of statistical independence is to aggregate multiple encounters from a single individual into "episodes" of illness, and is described in more detail below. Reliably automating this aggregation requires that every patient's records be uniquely identifiable.

To support the National Demonstration Bioterrorism Surveillance Program (NDP), we developed a system in which no PHI leaves the immediate control of the data provider, and only aggregate data is transferred to the datacenter [2,5]. Each data provider performs initial aggregation of the PHI within their own existing, secured data processing environment, producing data that is aggregated beyond the point where any individual patient is identifiable. Since data processing is distributed to the site of data collection rather than being performed at one central location, we describe this as a distributed processing surveillance system. Although this particular aspect of our work has briefly been mentioned in previous publications [1,2,4-6], we present it in greater detail here, because we believe that it represents a potentially valuable alternative surveillance system design option that deserves more explanation and wider debate than it has received to date.

## Discussion

The basic principle of distributed processing is simple. Rather than collecting all needed identifiable, individual PHI records centrally for statistical processing, all PHI is pre-processed remotely, and remains secured, under the direct control of the data provider. Only aggregate data are transferred to the central datacenter for additional statistical processing, signal detection, display and distribution. At an appropriate level of aggregation, the risk of inadvertent PHI disclosure becomes very small, and may prove acceptable to data custodians and to individual patients. Although this risk is never completely absent, it is certainly decreased in aggregate data, making this approach far more acceptable to data providers in our experience, than the more traditional approach of centralized collection of directly identifiable PHI.

### Centralized processing models for public health surveillance

Before describing our distributed system, we briefly review the more familiar model of centralized aggregation and processing of PHI for surveillance. In the more traditional type of system, individual patient records, often containing potentially identifiable information, such as date of birth and exact or approximate home address, are transferred, usually in electronic form, preferably through some secured method, to a central secured repository, where statistical tools can be used to develop and refine surveillance procedures. One of the main benefits of this data-processing model is that the software and statistical methods can be changed relatively easily to accommodate changes in requirements, because they only need to be changed at the one central location where analysis is taking place. As long as appropriate details have been captured for each individual encounter of interest, the raw data can be re-coded or manipulated in different ways. Only one suite of analysis code is needed, and because it is maintained at a single, central location, costs for upgrading and maintenance are small. Inadvertent disclosure of PHI is always a potential risk with centralized systems. Even where minimally identifiable data are stored in each record, the probability of being able to unambiguously identify an individual increases as multiple, potentially linkable records for that individual accrue over time.

### Distributed data processing by the NDP

Rather than gathering identifiable PHI information into a central repository for analysis, a distributed system moves some of the initial data processing, such as counting aggregated episodes of care (see below), to the site where the data is being collected. This aggregation minimizes the number of individuals who have access to PHI and diminishes the risk of inadvertent PHI disclosure from the surveillance system, while still allowing effective use of the information of interest. The focus of this report is on the model used to collect surveillance data while providing maximum protection for PHI, so the statistical methods we use in the NDP, which have been described elsewhere [4] are not discussed further here.

**Figure 1:**
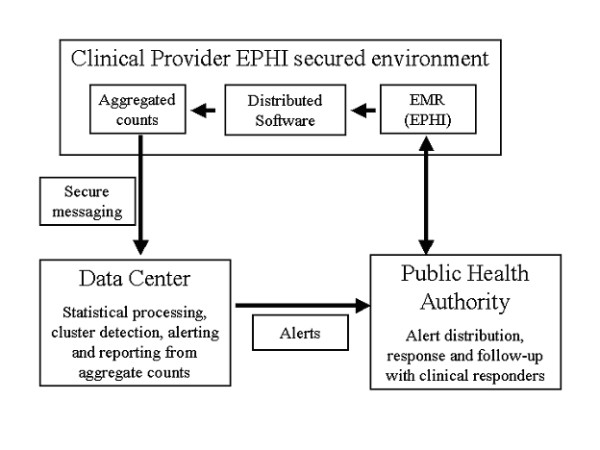
Distributed processing model and data flow.

Data flows for the NDP are illustrated in Figure 1. Data pre-processing, detection of repeated visits by the same patient for the same syndrome, and data aggregation is performed using a custom software package, written, maintained, and distributed by the NDP datacenter. Data providers maintain complete control of the security of their own PHI and also maintain control over the operation of the data processing software, which runs on one of their secured workstations. Since the pre-processing takes place within a secured environment under the control of the data provider, there is no need for the individual patient identifiers to be divulged to the datacenter. In the case of the NDP [5], the only data that is centrally collated consists of counts of the number of new episodes of specific syndromes over a defined time period (currently set at each 24 hour period ending at midnight), by geographic area (currently, 5-digit zip code area). More detailed definitions of "syndromes" and "new episodes" are provided below. Table 1 illustrates the data transferred from each data provider each day to the datacenter for statistical processing, reporting and alerting. Note that the although this data does not contain any obvious identifiers such as date of birth or gender, there is always a risk that a specific individual might be identifiable using additional data, and that this risk is greatest in zip codes with very small populations.

All source code required to build the data processing software is provided to the data provider at installation and whenever the software is updated, so that the local information services staff can check that there are no "backdoors" or other ways the distributed software could compromise the security of their systems. All information transferred to the datacenter is stored in text files (in XML format) and can be readily accessed by local staff to ensure that no PHI is being transmitted.

### Aggregation – definition of syndromes of interest

Participating data providers have near real-time ICD9 codes for every encounter, usually assigned by clinicians at the time of the encounter. Since much acute infectious disease manifests as broad suites of nonspecific symptoms, we monitor 13 syndromes – Respiratory, Lower gastro-intestinal (GI), Upper GI, Neurological, Botulism-like, Fever, Hemorrhagic, Skin lesions, Lymphatic, Rash, Shock-death, Influenza-like illness and SARS-like illness. All syndromes except Influenza-like illness and SARS-like illness were defined by a working group led by CDC and Department of Defense [7]. Individual ICD9 codes are used to aggregate encounters into one of these 13 syndromes. The definitions (ICD9 code lists) of 11 of these syndromes are available [7]. The definitions comprising the other two syndromes were developed in consultation with both CDC and the Massachusetts Department of Public Health.

### Encounters and episodes of a syndrome

Our surveillance algorithms [4] require statistically independent observations and are based on new episodes of syndromes. Our goal was to distinguish health care encounters that were related to ongoing care for any given episode of acute illness from the *initial *encounter that indicated the start of a new episode of a syndrome of interest. The derivation of the specific method for identifying first encounters for an episode of illness has been described in more detail elsewhere [1]. We define a new episode to begin at the first encounter after at least a 42-day encounter-free interval for that specific patient and that specific syndrome. If there has been any encounter for that specific syndrome for the same individual patient within the previous 42 days, the current encounter is regarded as part of the usual ongoing care for the original encounter that signalled the start of an episode of illness of that syndrome. The start of a new episode for a *different *syndrome can occur during ongoing encounters for any given specific syndrome – ongoing encounters during an episode are counted as new episodes only if they are *outside *(i.e. at least 42 days since the last encounter)of an existing episode of the matching syndrome. As will be described later, all ongoing encounters within any syndrome are recorded, and are visible through reports under the control of the data provider, but they do not contribute to the counts that are sent to the datacentre for analysis. All of this processing requires consistent and unique patient identifiers for all encounters. We use the local patient master index record number for this purpose in the software that we provide, but these identifiers are not required once the processing is complete, and they remain under the complete control of the providers.

### Standard input file formats

The distributed software requires the data providers to extract information about encounters of interest (daily, in our case) and convert it into the uniform format used by our distributed software. This kind of uniform representation is required for any multi-source surveillance system and is not peculiar to the distributed model we have adopted. In practice, we found that data providers could easily produce text files containing data as comma separated values in the format which we specified, and which the distributed software has been written to process. However, this requires dedicated programming effort that was supported with resources from the NDP grant.

### Data transfer

Our project receives support from the CDC, so we are required to comply with relevant CDC standards. Although the data being transferred to the datacenter is arguably not identifiable PHI because of the high level of aggregation, we use the Public Health Information Network Messaging System [8] (PHINMS), a freely available, secure, data transfer software suite developed by the CDC, to transfer aggregate data. A PHINMS server operates at the datacenter and each data provider operates a PHINMS client, using a security certificate supplied by the datacenter for encryption and authentication. PHINMS allows fully automated operation at both the datacenter and at each data provider. PHINMS communicates over an encrypted channel and usually requires no special modification to the data provider firewall, since it is only ever initiated by an outgoing request (the data provider always initiates the transfer of new data) and uses the same firewall port and protocol (SSL on port 443) as commercially encrypted services such as internet banking. PHINMS is reasonably robust to temporary connectivity problems, as it will try to resend all messages in the queue until they are delivered. Data transmission is one of the least problematic aspects of maintaining this system. We provide automatic installation software and it runs more or less instantaneously and transparently, without intervention in our experience. No training is needed as the process is fully automated.

### Data representation and application development language

All data is transferred to the datacenter in the form of eXtensible Markup Language (XML) since this is a flexible machine-readable representation and is easy to integrate with PHINMS. We used the Python [9] language for the development of the distributed software package. This choice was partially motivated by the fact that Python is an open-source language and thus freely distributable, partly by our very positive experience with Python as a general purpose application development language, and partially because in our experience, Python can be installed, and applications reliably run without any change to source code, on all common operating systems (including Linux, Unix, Macintosh and Windows), making it easy for the datacenter to provide support for systems other than Windows PC's. It is also a language with extensive support for standards such as XML, and securely encrypted internet connections. In addition, our existing web infrastructure has been built with the open-source Zope [10] web application framework, which is written mostly in Python.

### Reports available and benefits to data providers

A major design goal for our distributed software was that it should offer potentially useful functions for the data provider. This was motivated by our desire to encourage data providers to look at their own data in different ways that might not only help them manage the data more efficiently, but might also help them to more easily identify errors. In our experience, the task of maintaining a system like the one we have developed is far more attractive and interesting to the staff responsible at each participating institution if they gain some tangible, useful and immediate benefits. In addition, easy access to data flowing through our software is useful for ensuring transparency and to facilitate security auditing by each data provider.

The distributed software optionally creates reports that show one line of detailed information about each of the patient encounters that was counted for the aggregate data for each day's processing. These reports are termed "line lists" and were designed to support detailed reporting of encounter level data, so that a data provider can quickly make this information available in response to a public health need. Two versions are available, one with and one without the most specific identifying details, such as patient name and address. These standard line lists are used most often to support requests by public health agencies for additional information about the individual cases that contribute to clusters identified in the aggregate data. These lists are never transmitted to the datacenter but may be used to support public health officials investigating a potential event.

### Current capacity of the NDP to respond to public health needs

When unexpectedly high counts of particular syndromes are detected in geographically defined areas, the datacenter automatically generates electronic alerts, which are automatically routed to appropriate public health authorities. For example, in Massachusetts, electronic messages are automatically sent to the Massachusetts Alert Network within minutes of detection, where they are automatically and immediately forwarded to the appropriate Public Health personnel for follow up. Available alert delivery methods in the Massachusetts system range from email through to an automated telephone text-to-speech delivery system. Responders can configure the alert delivery method for each type of alert they have subscribed to. This alerting system is independent of our distributed system, but in practice, the ready availability of reports in electronic format containing both fully and partially identifiable clinical data for all cases comprising any particular period or syndrome makes the task of the clinical responder much simpler whenever a query is received from a public health official. Electronic reports, containing clinical information and optionally, full identifiers for all encounters can be generated as required, at the provider's site, from where they can immediately be made available to public health agencies. In the NDP's current operational mode (see Figure 1), a public health official calls a designated clinical responder to obtain this information.

The reports have one line per encounter and can be sorted and culled to create subset lists of only the cases contributing to a particular alert. The original, full line lists are created in two forms. The fully identified version contains all elements deemed of possible utility to the responder, namely: syndrome; date of encounter; whether the encounter was new or a follow-up to one occurring within the previous 6 weeks; date of the previous encounter, if any; date of birth; sex; town and zip code of residence; type of encounter (regular office visit, urgent care visit, phone call); temperature, if recorded; text corresponding to diagnostic codes assigned by the clinician; tests ordered, in broad categories; physician ID; and medical record number (Table 2). The "narrow" version, which contains fewer identifiers, provides each patient's five-year age group instead of date of birth and does not include the physician ID or medical record number (Table 3). At the provider's discretion, the clinical responder can provide the "narrow" list corresponding to the cases of interest to the public health department. If on this basis public health officials decide that further investigation is warranted, they can call the clinical provider and request a review of medical records, identifying the cases of interest by date and an index number (unique within date) in the narrow line list. The clinician finds the medical record number by looking up the date and index number in the wide line list and then accesses the record itself through the usual HMO-specific means. Resources to support clinical responders were provided through our NDP grant to participating data providers.

It would be straightforward to send detailed lists of encounters that are part of clusters directly to the relevant health department whenever the datacenter detects an event and sends an automated alert to a health department. We have not implemented this feature because all the participating health plans prefer to have an on-site clinical responder participate in the initial case evaluation with the public health agency. It would also be simple to allow designated public health personnel to initiate requests for specific line lists, even when no alert has occurred. Public health officials may, on occasion, wish to inspect the line lists to search for specific diagnoses that do not occur frequently enough to trigger an alert for their syndrome, but may be meaningful in the context of information that arises from other sources.

### Enhancing the utility of distributed data-processing for public health surveillance

Although not currently implemented in the NDP, it would be feasible to allow a remote user to perform ad-hoc queries on the encounter data maintained by the health plan. Examples of these queries include focused assessment of disease conditions affecting subsets of the population or specific diagnoses. This type of direct query capability is currently used at some of the same participating health plans to support the CDC's Vaccine Safety Datalink project [11], a surveillance system that supports post-marketing surveillance of vaccine safety [12].

### Current status, feasibility, advantages and generalisability

This distributed data model supports active surveillance and alerting of public health agencies in five states with 7 participating data providers. The system has proven to be workable, and it supports the syndromic surveillance needs of the participating health departments. There are fixed costs such as programming to produce the standard input files, installation and training, associated with adding each new data provider, so we have focussed our efforts on large group practices providing ambulatory care with substantial daily volumes of encounters, completely paperless electronic medical record systems, and substantial technical resources, since these enable us to capture large volumes of transactions with each installation. Relatively large numbers of encounters are needed to ensure that estimates from statistical modelling are robust. Applying a distributed architecture to surveillance from multiple smaller practices may enable appropriately large numbers of encounters to be gathered, but may prove infeasible because of costs and lack of appropriate internal technical support and because of heterogeneity in the way ICD9 codes are recorded and assigned by each data provider. Once the programming for standard input files is completed, installation and training take approximately one day total, usually spread out over the first two weeks. Nearly all problems are related to providers getting the standard file format contents exactly right, and to transferring these to the

The distributed architecture currently in use by the NDP allows clinical facilities to provide the aggregated information needed to support rapid and efficient syndromic surveillance, while maintaining control over the identifiable PHI and clinical data that supports this surveillance. The system provides support for the clinical providers to respond quickly to public health requests for detailed information when this is needed. In our experience, such requests involve only a tiny fraction of the data that would be transferred in a centralized surveillance model, providing adequate support for public health with minimal risk of inadvertent disclosure of identifiable PHI.

We believe this design, in which patients' clinical data remains with their own provider under most circumstances, while public health needs are still effectively met, conforms to the public's expectations, and so will be easier to justify if these surveillance systems come under public scrutiny. Many of the details of our approach are specific to the United States context, but the general principle of using distributed processing to minimise the risk of inadvertent PHI disclosure is of potential utility in other developed countries, although the specifics of our implementation may be less useful.

### Disadvantages compared with traditional approaches

The benefit of decreased risk of inadvertent PHI disclosure from our approach entails three principal disadvantages compared with routine, centralized collection of identifiable data. First, a clinical responder with access to the locally stored PHI data must be available to provide case level information when a cluster is detected. It would be technically straightforward to provide detailed information for relevant cases automatically when signals are detected. We deliberately did not implement this feature in the current system, since the participating health plans expressed a strong preference for direct involvement in this process.

The second disadvantage is the need to pre-specify the syndromes, age groups, and other data aggregation parameters in advance, since changing these requires the distribution of a new release of the aggregation software. In practice, we have addressed this by means of configuration data for syndrome categories read from a text file as the application loads, so the application code itself does not need alteration. This limitation could be largely overcome by creating a remote query capability to support ad hoc queries on identifiable data that remains in the control of the provider.

The third disadvantage is the technical challenge of maintaining distributed software that must reliably process data that the programmers are not permitted to examine. While the software can be exhaustively tested on synthesized data, we have occasionally encountered subtle problems arising from previously unnoticed errors in the input data. Our experience suggests that when writing this kind of distributed application, extensive effort must be devoted to detecting and clearly reporting errors in the input data before any processing takes place.

### Source code availability

An archive of Python source code for the distributed software will be made available by the corresponding author upon request. Unfortunately no resources are available to provide technical or other support outside the NDP.

## Conclusion

In summary, we have implemented a near real-time syndromic surveillance system that includes automated detection and reporting to public health agencies of clusters of illness that meet pre-specified criteria for unusualness [5]. This system uses a distributed architecture that allows the participating health care provider to maintain full control over potentially identifiable PHI and health encounter data. The distributed software loads simple text files that can be created from the data stored in virtually any proprietary EMR system. It sends summary data suitable for signal detection algorithms via a freely available messaging system, to a datacenter that can manipulate the aggregated information and combine it with data from other providers serving the same geographic region, and which automatically generates and sends alerts when unusual clusters of syndromes are identified. The distributed software also facilitates efficient access to fully identified patient information when needed for following up a potential event.

## Abbreviations

CDC: Centers for disease control and prevention

EMR: Electronic medical record

HIPAA: Health Insurance Portability and Accountability Act

GLMM: Generalised linear mixed model

ICD9: International classification of diseases revision 9

NDP: National Bioterrorism Syndromic Surveillance Demonstration Project

PC: Personal computer

PHI: Personal health information

PHINMS: Public Health Information Network Messaging System

SARS: Severe acute respiratory syndrome

SSL: Secure sockets layer

XML: eXtensible markup language

## Competing interests

The author(s) declare that they have no competing interests.

## Authors' contributions

RL wrote the first draft of the manuscript after extensive discussions with KY and RP. KY and RP both made substantial intellectual contributions during the evolution of the submitted manuscript. KY prepared Figure 1 and all of the tables.

## Pre-publication history

The pre-publication history for this paper can be accessed here:



## Figures and Tables

**Figure 2:**
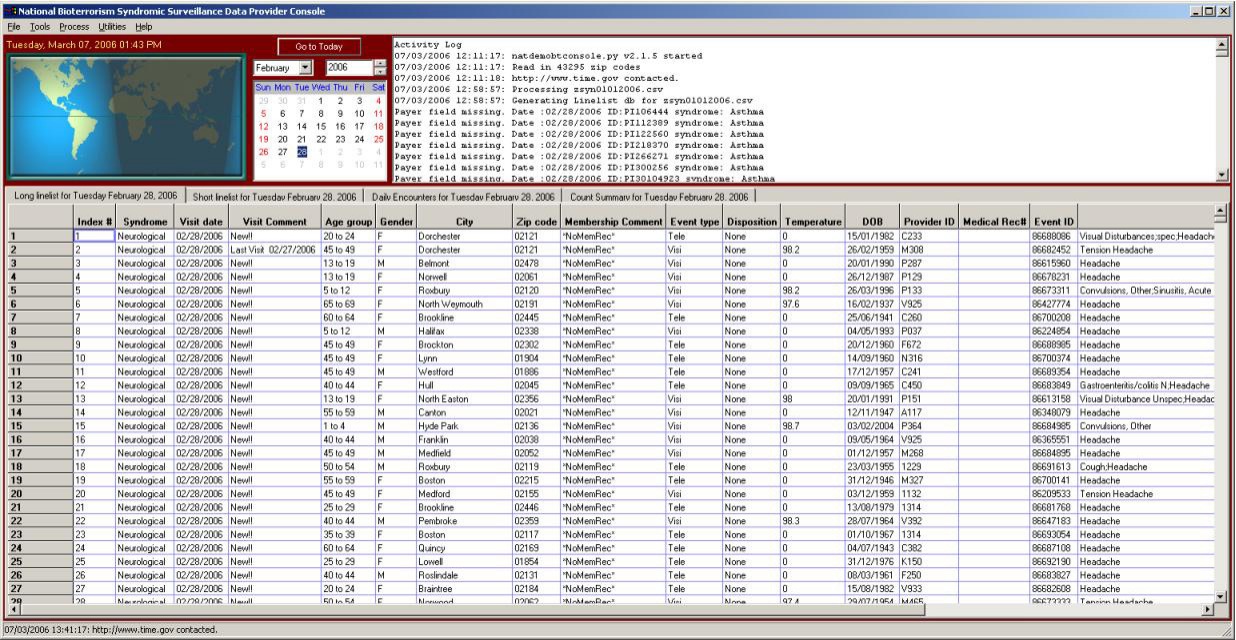
Distributed software screen, showing results (synthetic data) after daily processing of encounter records.

**Table 1:** Example of data elements transferred to the datacenter (synthetic data)

**Syndrome**	**Encounter date**	**Organization**	**Zip code**	**Town**	**Date of receipt**	**Count**
GI:lower	2/1/2006	KPNC	94565	Pittsburg CA	2/2/2006	7
GI:lower	2/1/2006	KPNC	95209	Stockton CA	2/2/2006	3
GI:lower	2/1/2006	KPNC	95632	Galt CA	2/2/2006	2
GI:lower	2/1/2006	KPNC	94509	Antioch CA	2/2/2006	6
GI:lower	2/1/2006	KPNC	94112	San Francisco CA	2/2/2006	3
GI:lower	2/1/2006	KPNC	95219	Stockton CA	2/2/2006	2
GI:lower	2/1/2006	KPNC	95122	San Jose CA	2/2/2006	3
GI:lower	2/1/2006	KPNC	94545	Hayward CA	2/2/2006	6
GI:lower	2/1/2006	KPNC	95825	Sacramento CA	2/2/2006	1
GI:lower	2/1/2006	KPNC	94806	San Pablo CA	2/2/2006	3

**Table 2:** Sample fully identified line list. (Synthetic data for purposes of illustration).

Index	Syndrome	Visit date	Visit Comment	Age group	Gender	City	Zip code	Event type	Temp	DOB	Provider ID	Medical Rec#	ICD9 codes	Tests ordered
18	Botulism-like	2/17/2006	New!!	35 to 39	M	Winchester	01890	Visit	0	6/3/1968	E263	234839	Visual Disturbances;spec	None
20	Botulism-like	2/17/2006	Last Visit 02/14/2006	5 to 12	F	Medford	02155	Visit	0	19/06/1999	E126	2623357	Diplopia	None
51	Fever	2/17/2006	Last Visit 01/31/2006	1 to 4	F	North Reading	01864	Visit	98.9	6/1/2003	11646	70462348	Viral Infections Unspecified;Unspecified Viral Infection	CSF
52	Fever	2/17/2006	New!!	60 to 64	M	Arlington	02476	Visit	97.4	14/01/1942	P036	70394015	Viral Infections Unspecified; Unspecified Viral Infection	None
107	GI:lower	2/17/2006	New!!	1 to 4	M	Ayer	01432	Tel. call	0	13/02/2003	C241	1696943	Abdominal Pain, unspecif.; Shortness Of Breath	None
108	GI:lower	2/17/2006	New!!	13 to 19	F	Burlington	01803	Visit	98.2	15/11/1991	11646	70463731	Gastroenteritis/colitis N	None
378	Neurological	2/17/2006	Last Visit 02/10/2006	40 to 44	M	Wilmington	01887	Tel. call	0	1/8/1961	18503	70458803	Convulsions, Other	None
379	Neurological	2/17/2006	New!!	55 to 59	M	Lowell	01854	Visit	0	29/05/1947	F609	1635454	Headache	None
411	Rash	2/17/2006	New!!	50 to 54	M	Weymouth	02188	Tel. call	0	15/11/1953	19217	70363320	Herpes Simplex Nos	None
434	Respiratory	2/17/2006	New!!	13 to 19	F	Hyde Park	02136	Visit	98.1	20/10/1988	18461	489441	Bronchitis Acute;Cough	None
435	Respiratory	2/17/2006	Last Visit 01/30/2006	40 to 44	F	Belmont	02478	Visit	0	6/9/1963	P326	392719	Mononucleosis, Infectious	None

**Note**: This report is kept by the health-care organization. Some or all records can be shared with the health department when a cluster requires follow-up of individual cases. Temp = patient temperature if recorded.

**Table 3:** Sample limited identity line list. (Synthetic data for purposes of illustration).

Index	Syndrome	Visit date	Visit comment	Age Group	Gender	City	Zip code	Event type	Temp	ICD9 codes	Tests ordered
18	Botulism-like	2/17/2006	New!!	35 to 39	M	Winchester	01890	Visit	0	Visual Disturbances;spec	None
20	Botulism-like	2/17/2006	Last Visit 02/14/2006	5 to 12	F	Medford	02155	Visit	0	Diplopia	None
51	Fever	2/17/2006	Last Visit 01/31/2006	1 to 4	F	North Reading	01864	Visit	98.9	Viral Infections Unspecified; Unspecified Viral Infection	Other cultures
52	Fever	2/17/2006	New!!	60 to 64	M	Arlington	02476	Visit	97.4	Viral Infections Unspecified; Unspecified Viral Infection	None
107	GI:lower	2/17/2006	New!!	1 to 4	M	Ayer	01432	Tel. call	0	Abdominal Pain, unspecif.; Shortness of Breath	None
108	GI:lower	2/17/2006	New!!	13 to 19	F	Burlington	01803	Visit	98.2	Gastroenteritis/colitis N	None
378	Neurological	2/17/2006	Last Visit 02/10/2006	40 to 44	M	Wilmington	01887	Tel. call	0	Convulsions, Other	None
379	Neurological	2/17/2006	New!!	55 to 59	M	Lowell	01854	Visit	0	Headache	None
411	Rash	2/17/2006	New!!	50 to 54	M	Weymouth	02188	Tel. call	0	Herpes Simplex Nos	None
434	Respiratory	2/17/2006	New!!	13 to 19	F	Hyde Park	02136	Visit	98.1	Bronchitis Acute;Cough	None
435	Respiratory	2/17/2006	Last Visit 01/30/2006	40 to 44	F	Belmont	02478	Visit	0	Mononucleosis, Infectious	None
436	Respiratory	2/17/2006	New!!	80+	M	Middleton	01949	Tel. call	0	Strep Sore Throat	None

**Note**: These can be shared with a health department to determine whether it is worthwhile to follow up individual cases, in which case full identifiers can also be provided. Temp = patient temperature if recorded

## References

[B1] Lazarus R, Kleinman KP, Dashevsky I, Platt R, Alfred DeMaria (2001). Using automated medical records for rapid identification of illness syndromes (syndromic surveillance): the example of lower respiratory infection. BMC Public Health.

[B2] Yih WK, Caldwell B, Harmon R, Kleinman K, Lazarus R, Nelson A, Richter B, Ritzwoller D, Sherwood E, Platt R, J. Nordin, B. Rehm (2004). National Bioterrorism Syndromic Surveillance Demonstration Program. Morbidity Mortality Weekly Report Supplement.

[B3] US Department of Health & Human Services Health Insurance Portability and Accountability Act. http://www.hhs.gov/ocr/hipaa/.

[B4] Kleinman K, Lazarus R, Platt R (2004). A generalized linear mixed models approach for detecting incident clusters of disease in small areas, with an application to biological terrorism. American Journal of Epidemiology.

[B5] Platt R, Bocchino C, Caldwell B, Harmon R, Kleinman K, Lazarus R, Nelson AF, Nordin JD, Ritzwoller DP (2003). Syndromic surveillance using minimum transfer of identifiable data: the example of the National Bioterrorism Syndromic Surveillance Demonstration Program. Journal of Urban Health.

[B6] Lazarus R, Kleinman K, Dashevsky I, Adams C, Kludt P, Alfred DeMaria J, Platt R (2002). Use of automated ambulatory-care encounter records for detection of acute illness clusters, including potential bioterrorism events. Emerging Infectious Diseases.

[B7] CDC (2005). Syndrome Definitions for Diseases Associated with Critical Bioterrorism-associated Agents.

[B8] CDC (2006). Public Health Information Network Messaging System.

[B9] van Rossum G (2006). Python.

[B10] Lloyd B, Zope Corporation (1997). ZOPE Web Application Framework.

[B11] Baggs J, Lewis E, JH A, Berger N, Blake M, Butani AL, Canton F, Drew L, Fox R, Lee M, Malais D, Norman R, Riedlinger K, Shay D (2004). Distributed data model for the Vaccine Safety Datalink (VSD) project..

[B12] Centers for Disease Control Overview of Vaccine Safety. http://www.cdc.gov/nip/vacsafe/#VSD.

